# Copy Number Variation and Differential Expression of a Protective Endogenous Retrovirus in Sheep

**DOI:** 10.1371/journal.pone.0041965

**Published:** 2012-07-24

**Authors:** Barbara Viginier, Christine Dolmazon, Isabelle Lantier, Frédéric Lantier, Fabienne Archer, Caroline Leroux, Christophe Terzian

**Affiliations:** 1 UMR754 “Rétrovirus et Pathologie Comparée” INRA, Université Lyon 1, Université de Lyon, Lyon, France; 2 Ecole Pratique des Hautes Etudes, Paris, France; 3 INRA Centre de Tours, Nouzilly, France; Institut Pasteur, France

## Abstract

The Jaagsiekte sheep retrovirus exJSRV and its endogenous counterpart enJSRV co-exist in sheep. exJSRV, a betaretrovirus, is the etiological agent of ovine pulmonary adenocarcinoma, and it has been demonstrated *in vitro* that an enJSRV Gag variant bearing the R-to-W amino acid change at position 21 was able to block exJSRV budding from the cells, providing a potential protective role for the host. In this work, we developed a fast mutation detection assay based on the oligo ligation assay (OLA) that permits the quantification of the relative proportions of the R21 and W21 Gag variants present in individual genomes and in cDNA obtained from normal and exJSRV-induced lung tumors. We have shown that the W21/R21 ratio is variable within and between breeds. We also describe for the first time that putative protecting enJSRV variants were expressed in alveolar type II cells (AECII), the major target of exJSRV.

## Introduction

Endogenous retroviruses (ERVs) represent a class of retroviruses that are inherited through the germline of their hosts like cellular genes. ERVs represent a significant portion of the host genomes (i.e., 8% of the human genome) and are generally inactivated by point mutations and/or indels [1]. However, some of these endogenous retroviruses still encode functional retroviral proteins. Two main hypotheses have been proposed to understand the maintenance of the coding capacities of ERVs: (1) endogenous retroviral proteins are necessary for the replication of ERVs, and (2) endogenous retroviral proteins are beneficial to their hosts. Several important results have shed light on the benefit of ERV proteins to their hosts. One well-illustrated beneficial impact of ERVs concerns the protection of the host from infection by related exogenous retroviruses. Protection can occur at the entry level of exogenous retrovirus through receptor interference resulting from competition for receptor binding between the exogenous and endogenous envelopes [2]. Protection can also occur later during the replication cycle of the retrovirus, either before the integration of the retroviral genome into the host nucleus [3] or during the assembly of the retroviral particles [4].

The enJSRV/exJSRV retroviruses in sheep represent a powerful model for the study of interactions between an ERV, its exogenous counterpart and their host. The sheep genome contains approximately 30 copies of the endogenous retrovirus enJSRV, which is highly related to the exogenous betaretrovirus exJSRV (90%–98% identity at the amino acid level), the etiological agent of ovine pulmonary adenocarcinoma [5]–[8]. Among the enJSRVs, it has been shown that the enJS56A1 provirus was able to block the release of infectious JSRV particles when co-expressed in cultured cells, illustrating a novel mechanism of retroviral interference [9]. The determinant of this blocking process resides at the level of a single residue at position 21 in the Gag precursor protein. The arginine residue at position 21 (R21) of the exJSRV Gag sequence is replaced by a tryptophan (W21) in the enJSR56A1 Gag sequence. This single change confers to enJS56A1 the ability to block the normal trafficking of JSRV particles to the plasma membrane. A complete set of enJSRV proviruses was characterized from a genomic library for the Texel breed, cloned and sequenced, revealing the presence of a second provirus containing non-synonymous mutations inducing the R->W change; this provirus was named enJSRV20 [10]. Interestingly, phylogenetic analysis strongly suggested that these two protecting proviruses became fixed in the sheep during its domestication [10].

**Table 1:** List of breeds analyzed. IL16 to 63 are inbred lines derived from the *Pre-Alpes du Sud* French breed.

Breed		No. of individuals
IL16		4
IL19		2
IL36		3
IL39		4
IL63		4
Texel (Tex)		2
Ile de France (IdF)		2
Bleu de Maine (BdM)		2
Rouge de l’Ouest (RdO		2
Vendéen (Ven)		3
Solognot (Sol)		3
Pol Dorset (Pol)		3
Suffolk (Suf)		3
Thônes et Marthod (Tho)		2
Romane (Rom)		2

Nothing is known concerning the variation of W21 enJSRV between individuals and between breeds. Therefore, we screened 42 sheep genomes and quantified the presence of W21 using an approach based on the oligo ligation assay (OLA), also known as the fast mutation detection assay [11]. Moreover, one can ask if protection against sheep exJSRV infection by W21 enJSRV, which was shown *in vitro*, is also relevant *in vivo*. Hence, previous data have shown that the enJSRVs are predominantly expressed in the placenta, and exJSRV infects and replicates in AECII [8] but it has never been shown if W21 RNA is present or not in these target cells. We then quantified the relative level of the W21 variant RNAs in AECII derived from tumoral and non-tumoral lungs. Our results indicate that W21 is present at variable frequencies in the genomes of several breeds and is expressed in the lung, the target of JSRV-induced transformation. Furthermore, we observed that tumoral lung samples rarely express the W21 variant, and when expressed, that it is expressed at a low level compared to that the level in normal lung samples.

**Figure 1:**
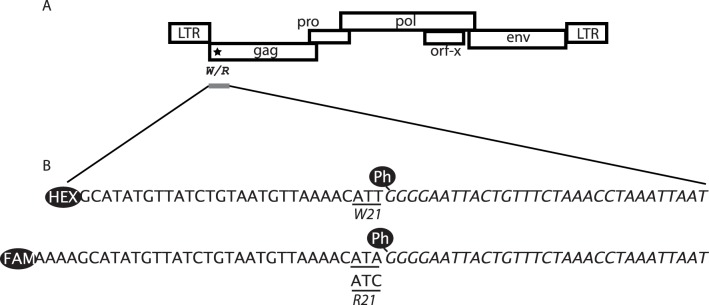
A. Canonical structure of the enJSRV provirus. The W/R variant at position 21 is indicated by a star. The 470 bp amplicon is represented by a gray bar. B. Ligation products obtained from the OLA detection assay of W21 and R21 variants. The sequence of the phosphate-modified downstream oligo is indicated in italics. The upstream oligo that detected the R21 to W21 mutation is labeled with HEX, whereas the two FAM-labeled oligos detected the R21 variant. Probes that were mismatched to the target by a single nucleotide at their junctions were not ligated. The ligation products were discriminated by size (60 nt for W21 coding sequence vs. 64 nt for R21 coding sequences) and color (HEX for W21 vs. FAM for R21). The W and R codons are underlined.

## Materials and Methods

### Ethics Statement

All animal experiments have been performed in compliance with our institutional and national guidelines in accordance with the European Community Council Directive 86/609/EEC. The INRA Tours ethics committee approved the experimental protocols.

**Figure 2:**
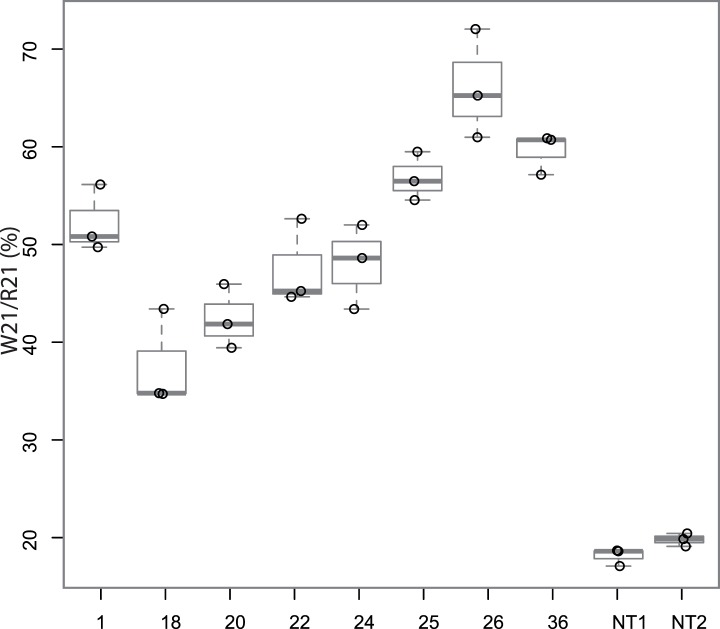
Boxplot representation of triplicate W21/R21 ratio measurements among randomly selected 10 samples. For each sample, W21/R21 measurement was repeated three times. Each dot represents a single measurement. Numbers correspond to the samples listed in Figure 1. NT1 and NT2 correspond to DNA extracted from two non-tumoral samples.

### Sample Collection

Forty-one DNA samples extracted from total blood have been collected from 10 ovine breeds and five inbred lines (IL) from the experimental flock maintained at the large animal facility, INRA, Tours Nouzilly France (Table 1). IL 16 to 63 are inbred lines derived from the Pre-Alpes breed. Fifteen samples of total lung DNA have been prepared from **7** Manech sheep from the French Basque Region that presented with respiratory symptoms and massive tumoral lesions upon gross examination and from 8 sheep of mixed breeds from a local slaughterhouse (Corbas, France) without macroscopic pulmonary lesions. Total DNA has been extracted using the Fast DNA kit as recommended by the manufacturer (Qbiogene, France). The exJSRV statuses of the sheep have been confirmed by histopathological examination to identify tumoral lesions (Anatomy and Pathological Cytology laboratory, Hospices Civils de Lyon, Pr Françoise Thivolet-Béjui) and by PCR detection of the exJSRV genome using exJSRV-specific primers targeting the 3'end of the *env* gene and the LTR (primer sequences available upon request). From the same 15 sheep, total RNA has been extracted using the standard RNeasy protocol (Qiagen, France) from primary AECII derived from non-tumoral (n = 5) or tumoral (n = 7) lung tissues as previously reported (Archer et al., 2007). The derivation of primary AECII failed for 3 tissues due to the poor conditions of the tissues. For each sample, 500 ng total RNA have been reverse-transcribed into cDNAs using random hexamers and M-MLV RT (Invitrogen) according to the manufacturer’s protocol and have been analyzed by PCR to determine their exJSRV status using specific primers.

**Figure 3:**
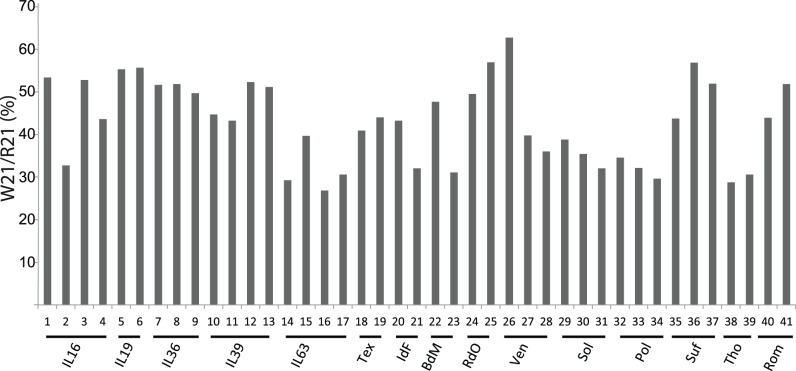
Distribution of W21 variants among 41 genomes from 10 breeds and 5 inbred lines. Individuals are numbered according to Table 1. The W21/R21 ratios are indicated as percentage.

### DNA and cDNA Gag Amplification

The PCRs were performed using DNAs or cDNAs and the primers enJSRV_482_ (5'-TCCTCGCCACTACTCTTATT-3') and enJSRV_953_ (5'-GGAGGGTCGCTTTTAACC-3') to amplify specifically the Gag region of enJSRV (Fig. 1A); the corresponding exJSRV region is not amplified using these same primers (not shown). The PCR reactions were performed in 50 µl volumes containing Taq polymerase buffer, 1.65 mM MgCl_2_, 200 µM each dNTP, 0.2 µM each primer, 100 to 500 ng of genomic DNA, and 1.25 U of Taq polymerase (Eurobio). The amplification conditions consisted of an initial 5 min denaturation at 95°C followed by 35 cycles of 94°C for 30 sec, 55°C for 30 sec, and 72°C for 1 min and a final extension of 10 min at 72°C. Positive and negative controls were based on PCR reactions performed using the penJS65A1 and pJS21 plasmids, which contain, respectively, enJSRV and exJSRV proviral sequences.

**Figure 4:**
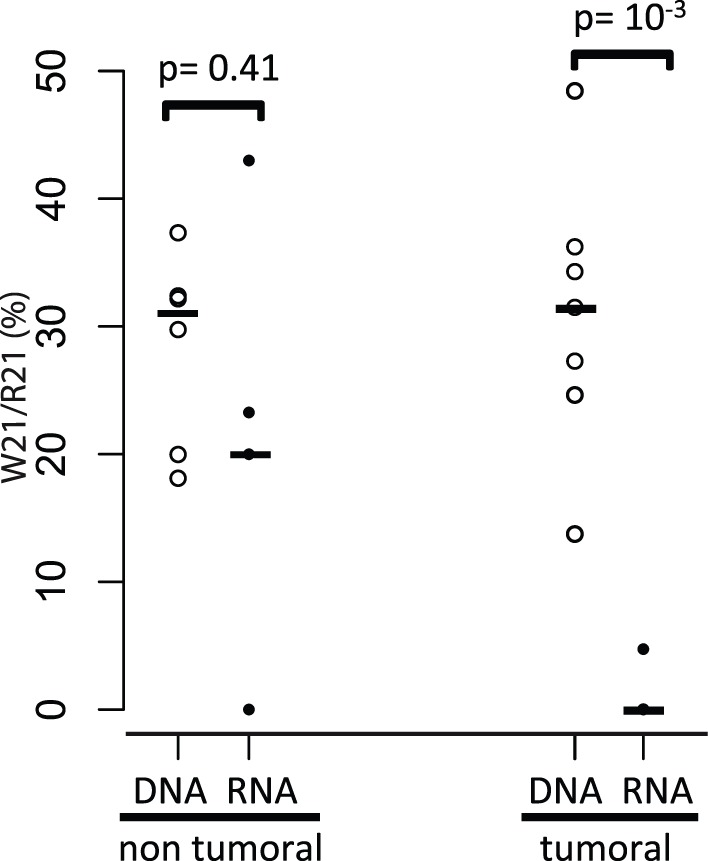
Distribution of the W21/R21 ratios between the DNA and RNA extracts from non-tumoral (respectively n = 6 and n = 5) and tumoral samples (respectively n = 7 and n = 7). Two Wilcoxon tests were used to compare the medians of W21/R21 ratios measured in DNA and RNA extracts from non-tumoral (p-value = 0.41, non significant) and tumoral samples (p-value = 0.001, highly significant).

### OLA Assay

OLA is a genotypic assay for the detection of known mutations based on the covalent joining of two adjacent oligonucleotides by a DNA ligase when they are hybridized to a DNA target [10]. For each reaction, 4 µl of purified amplicon, 4 U of thermostable *Thermus aquaticus* ligase (New England Biolabs, Beverly, MA), 5 nM upstream HEX or FAM-modified oligonucleotides (HEX-GCATATGTTATCTGTAATGTTAAAACATT, FAM-AAAAGCATATGTTATCTGTAATGTTAAAACATT, or FAM-AAAAGCATATGTTATCTGTAATGTTAAAACATA), and 5 nM downstream phospho-modified oligonucleotide (Ph-GGGGAATTACTGTTTCTAAACCTAAATTAAT) were added together in a 20 µl reaction (Fig. 1); the phospho-modification is necessary for ligation to occur. The ligation assay was performed using an initial 2 min denaturation at 94°C followed by 20 cycles at 94°C for 30 sec and 45°C for 3 min and a final denaturation of 10 min at 98°C. Only probes that matched to the target were ligated, resulting in a 60 nt HEX-labeled oligonucleotide for the W21 coding sequence, and in a 64 nt FAM labeled oligonucleotide for the R21 coding sequences (Fig. 1). Two microliters of a 1/10 dilution of the OLA products was mixed with 7.5 µl of deionized formamide and 0.5 µl of ROX-1000 size marker (PE Biosystems). The mixture was denatured at 95°C for 3 min and rapidly cooled on ice prior to analysis by a DNA sequencer (ADNid, France). The FAM and HEX peak heights values were determined by Gene Scan analysis (Applied Biosystems), and the data were analyzed using STR and 2.3.79 (Veterinary Genetics Laboratory, University of California, Davis, CA). The results have been expressed as the ratio of the HEX/FAM peak areas.

## Results and Discussion

### Validation of the OLA Assay to Estimate the Ratio of W21 vs. R21 Variants

Our first aim was to estimate the number of enJSRV proviruses coding the W21 Gag variant relative to the number of wild-type (R21) enJSRV proviruses. We performed a molecular assay based on the oligo ligation assay (OLA) of 41 genomes sampled from 10 sheep breeds and five inbred strains derived from the Pre-Alpes du Sud breed (Table 1). We developed an assay involving a PCR followed by a quantitative OLA that allowed the determination of the ratio of the copy numbers of the R21 and W21 enJSRV *gag* variants (Fig. 1). The PCR specifically amplifies a 470 bp region of the enJSRV *gag* gene encompassing the R21/W21 variant position. This specificity was provided by the reverse primer that targeted a segment within a variable region (VR1), which is highly divergent between exJSRV and enJSRV [12]. Four OLA probes were designed: the downstream phospho-oligonucleotide probe was common to the R21 and W21 *gag* sequences, whereas the three labeled upstream oligonucleotides were specific for the mutations responsible for the R21 and W21 variants (Fig. 1). Ligation only occurred when there was a perfect match between the upstream and downstream oligonucleotides. Labeled ligation products were then identified based on their fluorescent dyes and their sizes: 60 nt for W21 *vs.* 64 nt for R21 (Fig. 1).

The repeatability of the estimation of the ratio of W21 to R21 variants was tested in two steps. First, the penJS56A1 plasmid [6] containing the W21 variant was diluted to 0%, 20%, 50% and 100% *vs*. the pJaag101 plasmid containing the R21 variant; for each dilution, a PCR followed by the OLA was then performed twice on two replicates, and two curves (one for each replicate) were obtained by plotting the HEX/FAM peak area ratios *vs.* the serial dilutions. The two corresponding R-squared values were 0.96 and 0.99, reflecting the very high repeatability of the assay and the good prediction values of the curves (F-statistics and p-values = 10^−6^ and 10^−8^, respectively). Thus, the standard curve allowed intrapolation of the W21/R21 ratio for test specimens using the measured HEX/FAM ratio. Second, PCRs were performed on genomic and cDNA samples. Ten amplicons were randomly chosen, and OLAs were performed three times on each amplicon. The W21/R21 ratio was estimated using the previously constructed standard curves (Fig. 2), and the high repeatability of the W21/R21 measurement was confirmed by the non-parametric Friedman test (p-value = 0.41), indicating no significant difference between the triplicate measurements from the same sample. The OLA was then performed once for the 41 genomic samples from the breeds and twice for the others samples; in the latter case, the means of the two measurements were used.

### W21 Gag Variant is Present in the Genomes of Many Ovine Breeds

Our results indicate that the W21/R21 ratios are significantly different between ovine breeds (Kruskall-Wallis test, p = 0.01). This result suggests that the R21 and/or W21 enJSRV copy number varies significantly from breed to breed (Fig. 3). Interestingly, the W21/R21 ratio varies between individuals from the same inbred line (e.g., IL16) (Fig. 3), suggesting ongoing proviral amplification. We determined by Southern blotting that the enJSRV proviral copy number varies between but also within the IL16, 19, 36, 39 and 63 inbred lines (data not shown). Likewise, it was recently shown that enJS65A1 copy number can vary 5-fold between several individuals from Texel [13]. This result implies that the two enJS65A1 copies among the 30 enJSRV copies described in a single Texel individual by Arnaud et al. [10] are not representative of Texel individuals. Thanks to the OLA approach, we are now able to estimate the R21/W21 ratio in a large sample of individuals, and do not restrict our screen to enJS65A1 but consider all enJSRV types coding the W21 Gag mutant.

We then tested the hypothesis that a significant level of W21 Gag could provide a selective advantage for the hosts. Thus, we looked for the presence of W21 *gag* expression in AECII, the primary target of exJSRV together with Clara cells in the lung.

### The W21 Gag Expression Level in Sheep Lung Cells Depends on the Tumoral Status of the Sample

We compared the W21/R21 variant frequencies in DNA extracted from the 15 studied lungs (7 tumoral and 8 non-tumoral tissues) and, when available, *i.e.,* for 12 of the 15 animals, from the DNA from the corresponding *in vitro*-derived primary AECII (5 tumoral and 7 non-tumoral), established as previously reported [14]. All the tumoral alveolar type II cells harbored the JSRV provirus genome, while the non tumoral cell lines were negative [14]. We first estimated the W21/R21 ratios in the genomic DNA. The W21/R21 ratio varied from 13 to 48%, and the medians from the tumoral (T) and non-tumoral (NT) genomic samples were not significantly different (Wilcoxon test, p-value = 0.8). However, we observed a significant difference when we compared the medians between genomic DNA and cDNA extracted from tumoral and non-tumoral samples (Fig. 4): the median of W21/R21 cDNA (RNA) ratio was significantly lower than the median of W21/R21 genomic copies (DNA) in tumoral samples, whereas such a difference did not exist in non-tumoral samples (Fig. 4). This result indicates that the expression of W21 *gag* was null or lower in tumoral AECII and lung tissues than in non-tumoral samples. Whether the absence of W21 expression favors *in vivo* the replication of exJSRV could not be assessed in this study. However, it is known that the site of proviral integration can affect the expression by mechanisms that involve local epigenetic modifications, as described for position effects on the expression of transgenic integrants [15]. Comparing the W21 proviral insertion sites between infected sheep that develop a JSRV-induced lung adenocarcinoma and infected sheep that do not develop this adenocarcinoma would help us to answer to this question.

### Conclusions

In this study using a rapid, discriminative and quantitative molecular technique, we have shown that the W21/R21 ratio varies between individuals from different breeds. Moreover, our results indicate for the first time that W21 and R21 enJSRV are expressed in normal lung tissues and that W21 is not expressed or is expressed at a low level in tumor-derived AECIIs. Our present study did not demonstrate that the absence of W21 *gag* RNA (and therefore W21 Gag proteins) was the causal factor in the development of tumoral cells, but this study does provide new insights into the putative protective role of some endogenous retroviruses against infection by their exogenous counterparts.
